# Transmission of viruses via our microbiomes

**DOI:** 10.1186/s40168-016-0212-z

**Published:** 2016-12-02

**Authors:** Melissa Ly, Marcus B. Jones, Shira R. Abeles, Tasha M. Santiago-Rodriguez, Jonathan Gao, Ivan C. Chan, Chandrabali Ghose, David T. Pride

**Affiliations:** 1 Department of Pathology, University of California, San Diego, 92093 USA; 2 Human Longevity, Inc, San Diego, CA 92121 USA; 3 Department of Medicine, University of California, San Diego, 92093 USA; 4 Bioharmony Therapeutics Inc, New York, NY 10065 USA

**Keywords:** Saliva, Gut, Microbiome, Microbiota, Antibiotic perturbations, Antibiotic courses, Antibiotics, Virus, Virus transmission, Virome, Bacteriophage

## Abstract

**Background:**

Bacteria inhabiting the human body have important roles in a number of physiological processes and are known to be shared amongst genetically-related individuals. Far less is known about viruses inhabiting the human body, but their ecology suggests they may be shared between close contacts.

**Results:**

Here, we report the ecology of viruses in the guts and mouths of a cohort and demonstrate that substantial numbers of gut and oral viruses were shared amongst genetically unrelated, cohabitating individuals. Most of these viruses were bacteriophages, and each individual had distinct oral and gut viral ecology from their housemates despite the fact that some of their bacteriophages were shared. The distribution of bacteriophages over time within households indicated that they were frequently transmitted between the microbiomes of household contacts.

**Conclusions:**

Because bacteriophages may shape human oral and gut bacterial ecology, their transmission to household contacts suggests they could have substantial roles in shaping the microbiota within a household.

**Electronic supplementary material:**

The online version of this article (doi:10.1186/s40168-016-0212-z) contains supplementary material, which is available to authorized users.

## Background

Emerging data suggest that viruses are integral members of the human microbiome, inhabiting the surfaces of the skin [1], mouth [2], gut [3], and urinary tract [4]. Many of these viruses are bacteriophages that infect the numerous bacteria that also inhabit these surfaces, and may have antagonistic relationships (where they frequently kill their hosts) or mutualistic relationships (where they integrate into their host’s genome and potentially provide beneficial gene functions) with their host bacteria. Further study of these viromes has demonstrated that these bacteriophages carry numerous gene functions including complement and immunoglobulin degradation [2], and platelet binding [5] among other gene functions that might provide significant benefits to their bacterial hosts. Whether they kill their hosts rapidly or provide selective advantages to their hosts through the process of transduction, bacteriophages inhabiting the human virome may have a significant capacity to shape our cellular microbiomes.

How the microbiome responds to perturbations such as antibiotics may determine our susceptibility to pathogen colonization and/or to the development of certain diseases [6–8]. Viral communities also respond to perturbations, although in different ways than might be predicted based on the responses of their bacterial hosts. The gut viromes of mice and humans have more homologues to genes involved in antibiotic resistance after antibiotic perturbations [9, 10], but a recent study indicates that most of these genes might not be involved in antibiotic resistance [11]. Viral communities do not appear to have altered diversity in the long-term in response to antimicrobial therapy, potentially because viruses that exit the community after perturbations are replaced by other eukaryotic viruses [10].

The finding that bacteriophages are highly prevalent members of the human microbiome led to the hypothesis that viruses were transient in the human microbiome [2, 12], as they likely identified their hosts rapidly, killed them, and spread their progeny to other susceptible hosts. Recent studies now have shown that bacteriophage members of the oral [12] and gut [3] microbiomes can be highly persistent. That persistence can have consequences, as one study suggests that phages may be shared amongst close contacts [13]. If these phages can be transmitted between close contacts, the persistence provides further opportunities for the dissemination of gene functions such as complement or immunoglobulin degradation carried in human viral communities.

In this study, we recruited and sampled the saliva and feces of a cohort of genetically unrelated individuals from different households to discern whether viral members of the human microbiome may be transmitted between subjects as a result of close contact. We gave an antibiotic to one household member and placebo to the other household member to decipher whether the use of antibiotic perturbations may affect the direction of the sharing of bacteriophages in each household.

## Results

### Study cohort

We recruited a cohort of 20 subjects and sampled their saliva and feces over a 6-month period. Of the 20 subjects enrolled, there were eight separate households consisting of two individuals and four separate controls not enrolled with a housemate (Additional file 1: Table S1). In each household, one individual received treatment with an antibiotic (azithromycin or amoxicillin) for 7 days and the other individual received a placebo (vitamin C). The four separate controls did not receive any therapy. Each subject was sampled on day 0 (day prior to antibiotics), day 3 (on the third day of antibiotics), day 7, week 8, and at 6 months.

### Individual-specific patterns of viruses in the gut and mouth

We isolated viruses from all 97 fecal and 95 saliva samples according to our previously described protocols [2], which involved sequential filtering to remove cellular debris, CsCl density gradient ultracentrifugation, and DNA extraction from intact virions. Resulting DNA was sequenced using Semiconductor Sequencing [14] for a total of 118,527,761 reads with a mean length of 215 nucleotides. There were 63,092,083 fecal reads with a mean GC content of 39.9% and 55,435,678 salivary reads with a mean GC content of 43.0%. We sequenced an average of 5,926,388 reads per subject and an average of 1,234,664 reads per each time point.

**Fig. 1:**
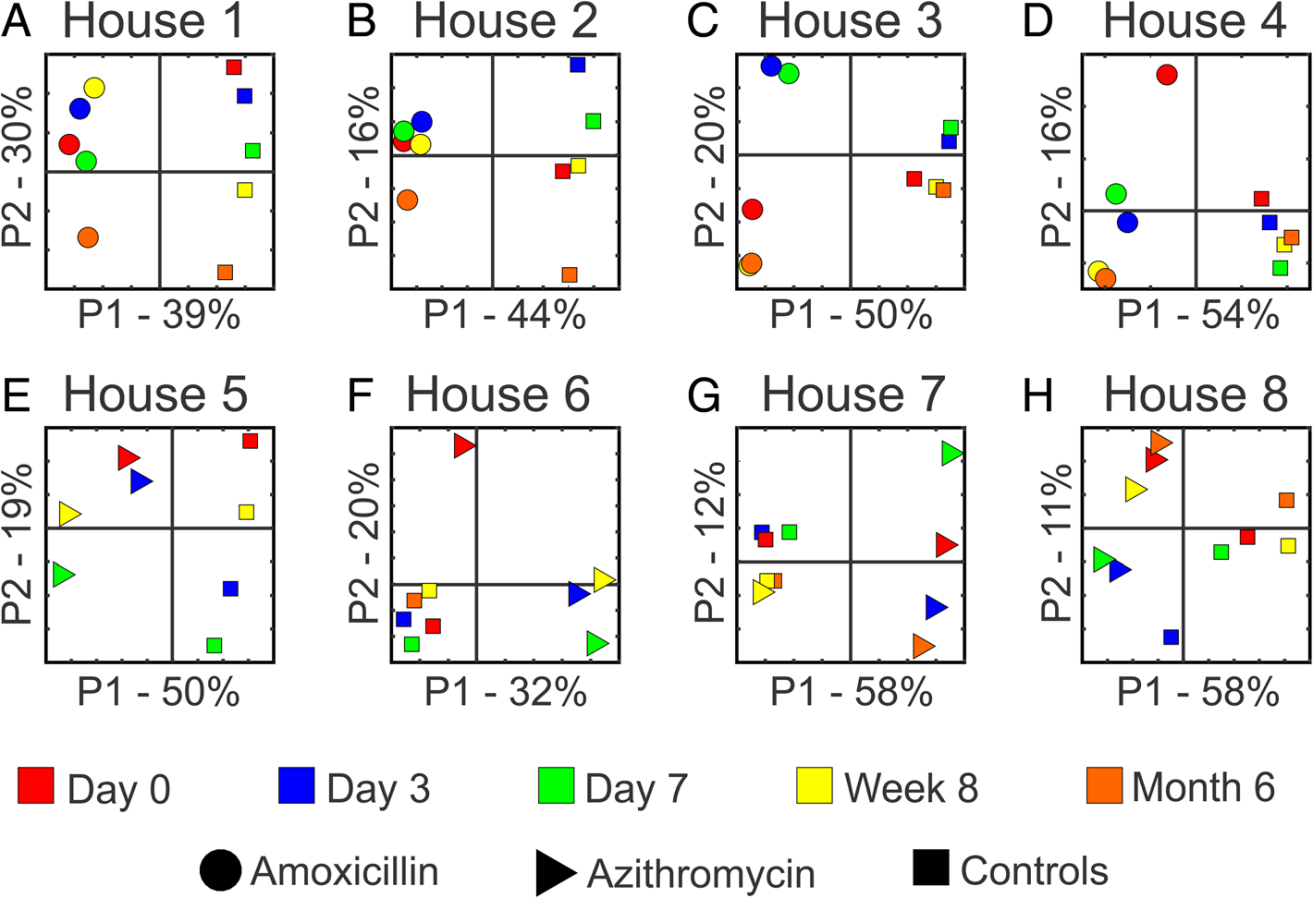
Principal coordinates analysis of beta diversity present in the feces of all households. Panels **a**–**h** represent households 1–8, respectively. Subjects who received placebo are represented by *squares*, amoxicillin are represented by *circles*, and azithromycin are represented by *triangles*. Specimens collected from day 0 are represented in *red*, day 3 in *blue*, day 7 in *green*, week 8 in *yellow*, and month 6 in *orange*

We characterized the ecology of the viruses in each of the eight households to decipher whether the individuals in each household could be distinguished based on their fecal or oral viruses and whether the use of antibiotics might disrupt individual-specific patterns of viruses within each household. Using principal coordinates analysis (PCOA) to visualize the beta diversity between household members, each individual could be distinguished based on their gut (Fig. 1) and oral viromes (Additional file 2: Figure S1). These individual-specific patterns were not disrupted by the use of either amoxicillin (Panels A–D) or azithromycin (Panels E–H). The individual-specific patterns were significant in the feces of 13/16 subjects and in the saliva of 7/16 subjects when using a permutation test to compare the viruses within an individual over time with that of their housemates (Table 1).

**Table 1:** Viral homologues within and between individuals

House	Subject	Percent homologous within subjects^a^	Percent homologous between subjects^a^	*p* value^b^
Feces				
House 1	CA05	73.55 ± 6.33	14.27 ± 5.13	*<0.0001*
	CA06	51.91 ± 11.30	12.48 ± 7.15	*0.0058*
House 2	CA13	61.49 ± 14.60	8.27 ± 6.01	*<0.0001*
	CA14	44.27 ± 20.86	2.96 ± 2.68	*<0.0001*
House 3	CA23	45.23 ± 19.70	3.23 ± 4.12	*<0.0001*
	CA24	43.87 ± 26.21	3.76 ± 4.56	*0.0009*
House 4	CA27	35.38 ± 29.00	3.61 ± 6.56	*0.0550*
	CA28	36.64 ± 23.35	1.47 ± 3.11	*0.0007*
House 5	CA11	20.35 ± 21.07	1.00 ± 2.07	0.0646
	CA12	27.99 ± 28.24	1.61 ± 2.94	0.0893
House 6	CA15	8.06 ± 11.04	3.87 ± 3.81	0.2800
	CA16	51.27 ± 6.92	3.30 ± 3.71	*<0.0001*
House 7	CA39	47.19 ± 14.19	10.20 ± 5.12	*0.0100*
	CA40	68.41 ± 3.30	11.35 ± 11.18	*<0.0001*
House 8	CA47	57.75 ± 5.80	14.74 ± 6.56	*<0.0001*
	CA48	60.67 ± 8.20	16.84 ± 7.64	*<0.0001*
Saliva				
House 1	CA05	14.11 ± 8.17	4.86 ± 3.71	0.1100
	CA06	28.25 ± 17.26	5.79 ± 5.90	0.1000
House 2	CA13	39.51 ± 14.08	3.57 ± 3.89	*0.0001*
	CA14	19.19 ± 17.37	6.24 ± 6.27	0.1635
House 3	CA23	26.85 ± 13.38	4.86 ± 6.57	*0.0459*
	CA24	29.54 ± 9.62	4.29 ± 5.86	*0.0099*
House 4	CA27	24.03 ± 9.35	11.35 ± 9.14	0.1798
	CA28	38.60 ± 26.63	16.75 ± 16.43	0.1275
House 5	CA11	29.57 ± 24.08	7.50 ± 5.85	0.1552
	CA12	31.71 ± 26.37	8.75 ± 7.28	0.1579
House 6	CA15	24.33 ± 22.43	8.59 ± 9.49	0.1438
	CA16	14.56 ± 18.61	7.09 ± 7.79	0.2335
House 7	CA39	9.51 ± 13.11	0.34 ± 1.19	*0.0505*
	CA40	47.95 ± 5.12	0.24 ± 0.45	*<0.0001*
House 8	CA47	35.80 ± 15.61	9.77 ± 8.34	*0.0529*
	CA48	39.48 ± 9.52	14.17 ± 8.09	*0.0211*

^a^Based on the mean of 10,000 iterations. Per iteration, 10,000 random contigs were sampled

^b^Empirical *p* value based on the fraction of times the estimated percent homologous reads within each subject exceeds that for different subjects. *p* values ≤0.05 are represented in italics

### Household-specific patterns of viruses in the gut and mouth

**Fig. 2:**
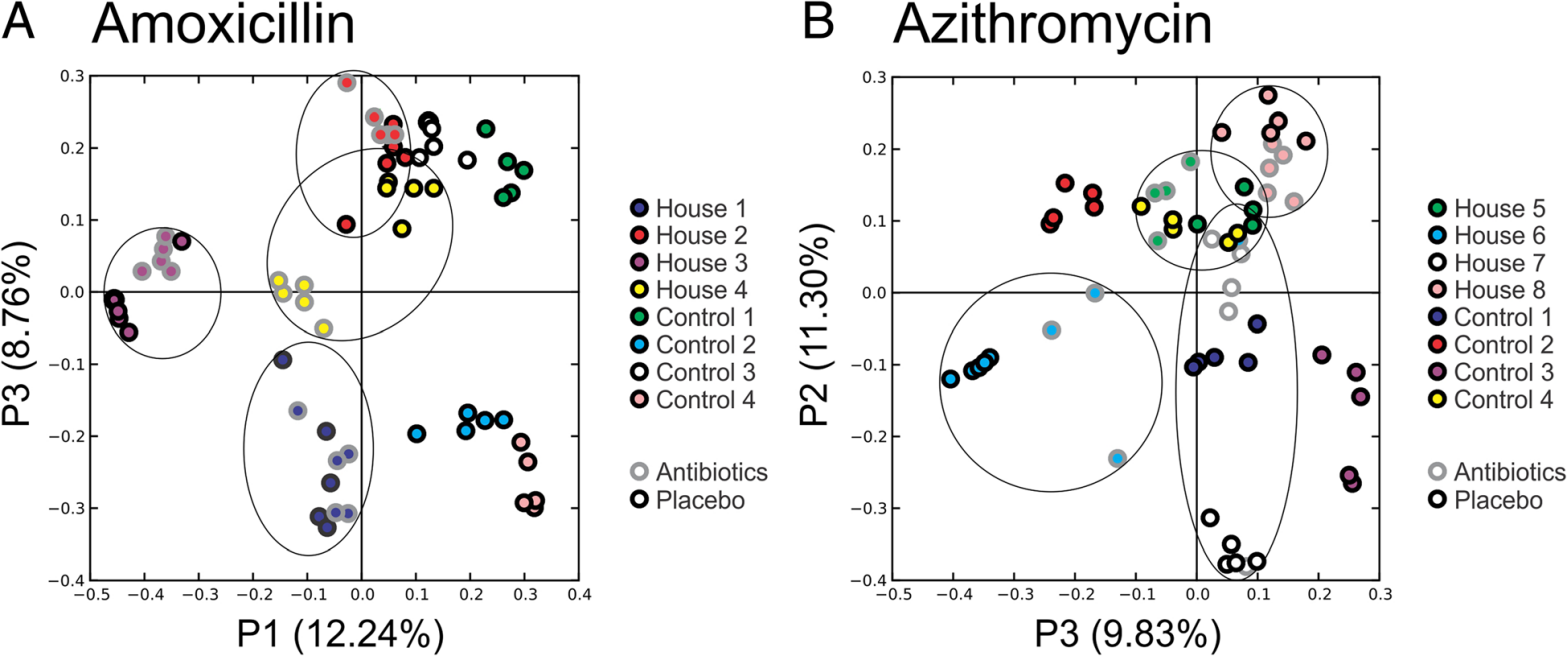
Principal coordinates analysis of beta diversity present in the feces of all subjects. Panel **a** represents subjects who received amoxicillin, their housemates who received a placebo and control subjects who received no therapy. Panel **b** represents subjects who received azithromycin, their housemates who received a placebo and control subjects who received no therapy. Controls who received placebo or no therapy are demonstrated by *black outer circles* and subjects who received antibiotics are represented by *gray outer circles*. Outlines are drawn around all time points representing individual households

We also tested whether household-specific patterns of viral ecology may exist in the cohort in addition to individual-specific patterns of viruses we observed (Fig. 1). We utilized PCOA to compare all subjects in households that received amoxicillin or azithromycin and observed patterns that suggested shared fecal viral ecology within the households for each household evaluated (Fig. 2). These patterns observed were statistically significant in the feces for five out of eight households as was observed through permutation analysis comparing the shared viral ecology within a household with the shared viral ecology between different households (Additional file 1: Table S2). Similar patterns were observed in the mouths of each of the household members, but were only significant in three out of eight households (Additional file 1: Table S2 and Additional file 2: Figure S2).

### Household viruses are persistent and shared

A prior study indicated that oral bacteriophages are highly persistent and that the gut viruses of a single individual were persistent over time [13]. We evaluated the persistence of viruses in our cohort over time to determine whether their persistence may be altered by the use of antibiotics. We found that fecal viruses were highly persistent with greater than 70% found at least 4 days apart, and 30% identifiable at 5 to 6 months (Additional file 2: Figure S3). There was no identifiable effect the antibiotics had on the persistence of phages, as similar patterns were observed in amoxicillin and azithromycin households that were observed in the controls who took no therapy. The levels of persistence in the gut far exceeded those observed in the mouth (Additional file 2: Figure S4).

Because we found patterns that suggested shared household viral ecology (Fig. 2), we quantified the proportions of shared viruses between the members of each household. We found similar patterns of shared viruses in the gut (Additional file 2: Figure S5, Panel A), and in the mouth (Panel B). There were generally more shared viruses within each household in the gut than were found in saliva, but the difference did not meet statistical significance (Additional file 2: Figure S6). The majority of the viruses that were not shared in the guts of housemates were found in those individuals who took a placebo (Panel A), while roughly equal numbers of unique oral viruses in each household were found in subjects who received either an antibiotic or placebo (Panel B).

**Fig. 3:**
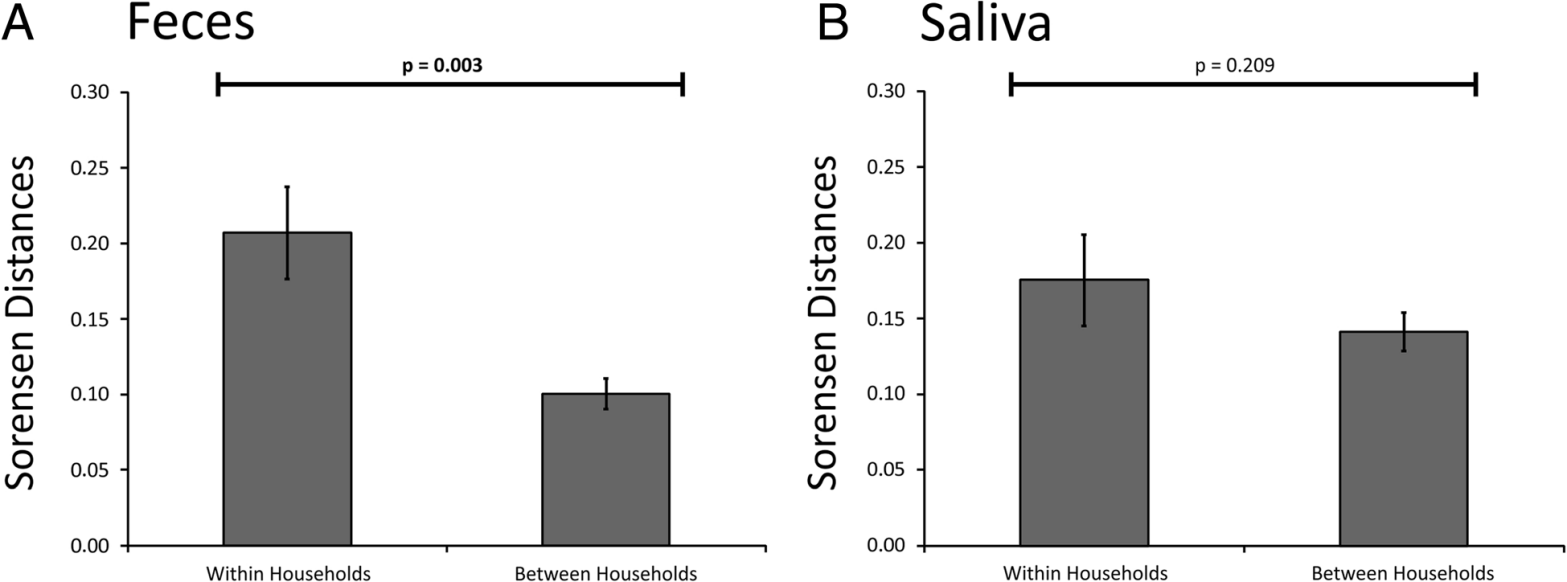
*Bar graphs* comparing Sorensen distances (± standard error) between subjects within a household and between subjects from different households for feces (panel **a**) and saliva (panel **b**). *p* values are represented *above* the bars

We also measured Sorensen distances between the individuals in each household and compared them with individuals from different households to decipher whether there were significant similarities between the individuals in each household that indicated that viruses were shared within a household. We measured Sorensen rather than Bray Curtis distances because Sorensen distances are useful for measuring similarity between viromes, while Bray Curtis distances measure dissimilarity. In the gut, we found significantly higher levels of similarity amongst household members than were found between individuals who resided in different households (Fig. 3, Panel A). We also identified higher similarity in the saliva for household members, but these results were not statistically significant (Panel B).

### Putative transmissions and directionality of transmissions in each household

We found bacteriophages that were shared in the mouth and the gut of housemates from all households (Fig. 3, Additional file 2: Figure S5, Figure S6). We examined the structure of some of these phages and found that some were present in both housemates on most days examined (Additional file 2: Figures S7–S10). We identified virulence factors within the genome structures of many of these phages, including an enterotoxin, toxin-antitoxin system, and platelet binding protein (Additional file 2: Figure S7), mucin and m23 peptidase (Additional file 2: Figure S8), and a beta-lactamase that could be involved in the degradation of beta-lactam antibiotics such as amoxicillin (Additional file 2: Figure S9). We also identified a phage with significant similarity to crAssphage [15] shared in the members of household 6 (Additional file 2: Figure S10). Finding genes potentially involved in antibiotic resistance in the structures of phage genomes shared between household contacts suggests that the sharing of these phages could be a means by which antibiotic resistance spreads through close contact with others.

**Fig. 4:**
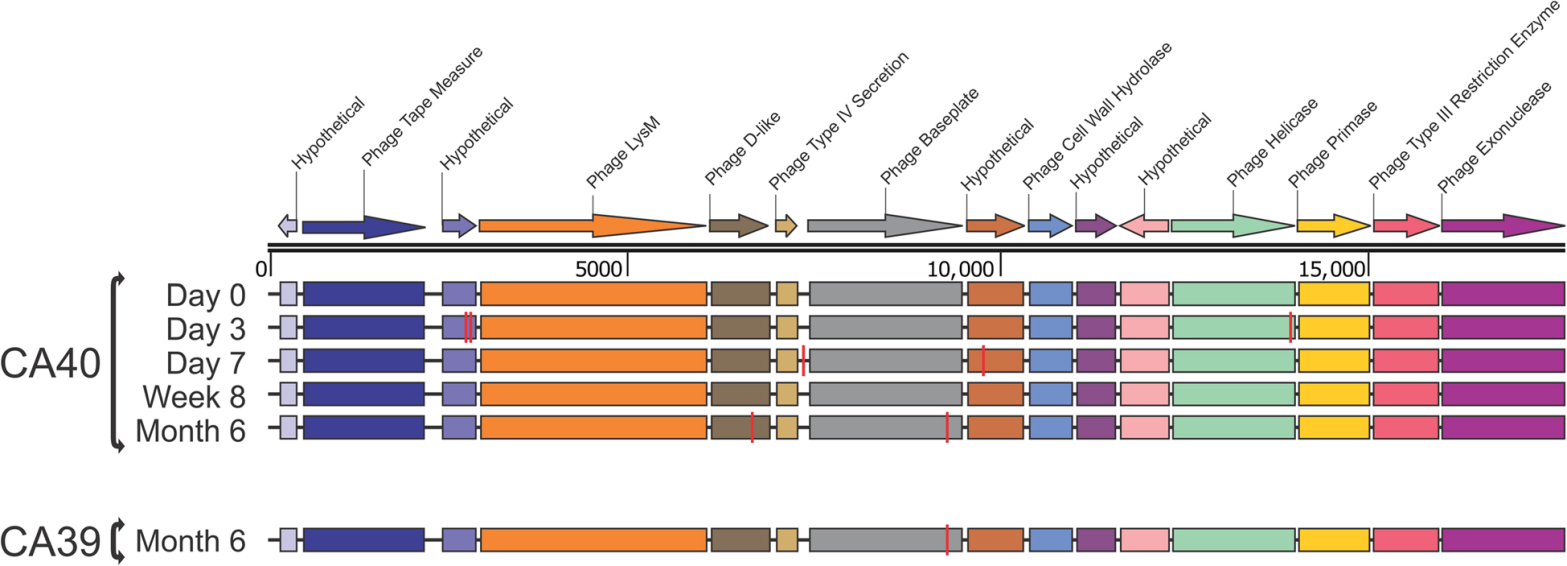
Diagram of contig71 assembled from Sanger sequences from all time points in subject CA40 and from the month 6 time point in subject CA39. The contig was not identified on days 0, 3, 7, or week 8 in subject CA39. Putative ORFs and their directions are indicated by the *arrows* at the *top* of the diagram. ORFs that had significant homologues (BLASTP E-score <10^−5^) are indicated by the text *above* each *arrow*. The location of polymorphisms (when compared to the day 0 time point in subject CA40) are indicated by *orange vertical lines*

**Fig. 5:**
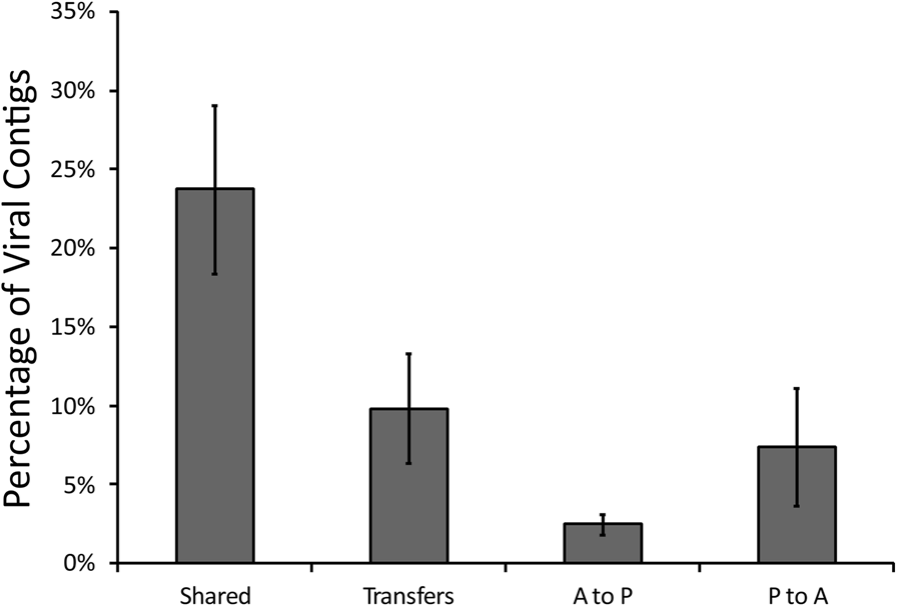
*Bar graph* representing the mean proportions of fecal viromes (± standard error) shared between housemates, putative transmissions between housemates, putative transmissions from subjects taking antibiotics to subjects taking placebo, and putative transmissions from subjects taking placebo to subjects taking antibiotics

We identified numerous fecal phages whose presence/absences on certain days between the housemates suggested that they had been transmitted from one household member to the other. For example, we could identify portions of a phage present in all days in the fecal viromes of one subject in household 7 (includes subjects CA39 and CA40), but only after 6 months in their housemate (Additional file 2: Figure S11). We verified the structure of this virus by PCR amplification and Sanger sequencing of its genome from all days in subject CA40 (Fig. 4). We attempted the same in the housemate (subject CA39), but the phage could only be amplified and sequenced from the 6-month time point. While there were a few polymorphisms in the phage over the course of time, the phage only had one polymorphism between subjects CA39 and CA40 at the 6-month time point. These data suggest that between week 8 and month 6, the phage was transmitted from subject CA40 to subject CA39. We characterized the presence/absence of viruses in all households over the course of the study and defined any virus that was present in one subject, absent in their housemate, and later appeared in at least two time points in the housemate as a putative transmission. Using these criteria, we found that of the 23.7 ± 0.05% of fecal viruses that were shared, 9.8 ± 0.03% had a pattern consistent with a putative transmission between housemates (Fig. 5). When examining the directionality of the putative transmissions, the majority (7.4 ± 0.04%) were consistent with transmission from the subject taking the placebo to the subject taking antibiotics, while the minority (2.4 ± 0.01%) went in the other direction. Similar results were identified in terms of the number of putative viral transmissions in the mouth, but there was little difference in the directionality of these transmissions between housemates (Additional file 2: Figure S12).

## Discussion

It has been well established that genetically related individuals and some spouses who share a household also share some portion of their microbiota [13, 16, 17]. Most of these studies have focused on the bacteria that inhabit the human microbiome, while ignoring the estimated 10^15^ viruses that inhabit the human body. We utilized CsCl density gradients to purify viruses which often results in highly purified virus fractions, but introduces biases when examining the relative abundances of some viruses [18]. Our data indicate that neither a genetic relationship nor a spousal relationship is required to share viruses, and that unrelated individuals who share a household also will share some proportion of their viruses. This sharing is not based on intimate contact, as both romantic couples and roommates were enrolled in this study and all of them shared viruses. Because six of the eight households enrolled were romantic couples, we could not discern statistically whether there were more shared viruses amongst the romantic couples; however, there was no observed trend of greater sharing of viruses amongst the households studied (Additional file 2: Figure S5). We did identify a non-significant trend of greater shared bacterial biota amongst romantic couples in a study using a larger cohort [19]. We did not control for how long the household members had lived together prior to the initiation of this study, but had households in this study who had lived together for as little as 1 month prior to this study, so it may be a matter of days or weeks before viruses are shared between household members. In general, there were greater levels of shared viruses found in the gut than in the mouths of our subjects, which we believe is related to the higher persistence of viruses in the gut and suggests less turnover and greater opportunities to share fecal viruses within a household. A prior study reported that individuals on similar diets converge upon similar gut virome phenotypes [3]. None of the subjects reported any changes to their diets during this study, but we did not monitor whether housemates shared meals, which could have resulted in greater shared virome contents.

Our finding of both virulence factors (Additional file 2: Figures S7 and S8) and an antibiotic resistance homologue (Additional file 2: Figure S9) within the structures of bacteriophages suggests that their sharing could have consequences for the microbiota of housemates to which these genes are shared. The sharing of antibiotic resistance genes such as a beta-lactamase could result in resilience to amoxicillin perturbations in the recipient; however, we have not demonstrated that the homologue is involved in antibiotic resistance. A recent study suggests that most of the homologues identified to antibiotic resistance genes in viromes may not be involved in antibiotic resistance; thus, functional assays need to be performed to demonstrate antibiotic resistance in viromes. While only about 10% of the shared viruses were identified as putative transmissions, the fact that we did not control for the length of time individuals had cohabited prior to the beginning of the study suggests a larger portion (about 15%) of the viruses may have been transmitted between subjects prior to the start of the study. A more controlled study where subjects are sampled both prior to and after they cohabit would be necessary to gain a more detailed understanding of the dynamics of viral transmissions between individuals.

Despite the use of antibiotics, we could still identify each subject based on their virome contents regardless of the time point studied. These results are similar to a prior study in which we noted that human oral viruses were highly persistent over time [12]. The use of relatively narrow spectrum oral antibiotics such as amoxicillin and azithromycin may have contributed to the stability observed in virome contents, as we observed much greater turnover in virome contents when broad spectrum intravenous antibiotics were used in a separate cohort [10]. We believe that there is a relatively stable core of viruses that inhabit an individual’s microbiome, and that there are other viruses more susceptible to being replaced as individuals are exposed to perturbations such as viruses transmitted from their close contacts. Further studies are necessary to demonstrate such a phenomenon.

## Conclusions

There now are quite a few studies characterizing the viruses that inhabit human body surfaces. Those studies have demonstrated that viral communities are highly diverse, carry substantial pathogenic gene functions, are populated by viruses that persist over time, and have individual- and body surface-specific ecology [1, 3–6, 12, 20]. Our data indicate that viral communities in the mouth and gut are readily shared within a household, likely through transmissions from one subject to another. While it is not clear whether most of the sharing is from direct viral transmissions rather than lysogen (bacteria with un-induced prophage) transmissions, the sharing results in viromes formed through interactions with individuals and their environments. We do not yet fully understand the extent to which bacteriophages in humans may help to shape the ecology of the bacteria in our microbiomes, but the data presented here suggest that our viruses not only have the capacity to shape the natural history of our microbiomes on multiple body surfaces, but also have the potential to shape the natural history of the microbiomes of our close contacts.

## Methods

### Cohort design

Sixteen subjects were enrolled in the study in pairs, with two individuals living in each household. An additional four individuals were enrolled without a housemate and received no therapy over the course of the 6-month study. Of the household pairs, four pairs were placed into the amoxicillin arm and four pairs were placed into the azithromycin arm. In each household, one subject received 7 days of an antibiotic and the other subject received 7 days of the placebo (vitamin C). The dose of amoxicillin was 500 mg twice daily, and the dose of vitamin C was 500 mg twice daily. The dose of azithromycin was 500 mg on the first day, and 250 mg daily thereafter (this dosing was used to be consistent with the commonly prescribed Z-Pak). In the azithromycin arm, the placebo was given at 500 mg once daily. Each subject enrolled donated saliva and feces on day 0 (day prior to antibiotics), day 3 (3 days after initiation of antibiotics), day 7, week 8, and month 6. Of the eight households enrolled, one of those households was lost to follow-up and did not provide specimens at the month 6 time point. Each subject provided at least 3 ml of unstimulated saliva. All subjects were encouraged to provide specimens in the AM prior to breakfast and freeze them at −20 °C prior to transporting on ice to the study site, where they were frozen at −80 °C until use in this study. Exclusion criteria included prior antibiotic use for 1 year prior to the initiation of the study, and preexisting medical conditions such as diabetes, inflammatory bowel disease, and organ transplantation that might result in significant immunosuppression. All subjects self-reported their health status and were genetically unrelated.

### Analysis of viromes

Fecal viromes were prepared by diluting 0.4 g of feces in 4 mL of SM buffer, vortexing for 40 min to separate viral particles, spun at ×4000*g* for 10 min to pellet the remaining solid material, and the supernatant was treated in an identical manner to saliva specimens. Saliva samples and fecal supernatants were filtered sequentially using 0.45 and 0.2 μm cellulose acetate filters (GE Healthcare Life Sciences) to remove cellular and other debris, and then purified on a cesium chloride gradient according to previously described protocols [2]. Only the fraction with a density corresponding to most known bacteriophages [21] was retained, further purified on Amicon YM-100 protein purification columns (Millipore, Inc.), treated with two units of DNase I, and subjected to lysis and DNA purification using the Qiagen UltraSens Virus kit (Qiagen). Recovered DNA was screened for the presence of contaminating bacterial nucleic acids by quantitative 16S rRNA gene PCR using primers 8 F (AGAGTTTGATCCTGGCTCAG) and 357R (CTGCTGCCTYCCGTA) in Power SYBR Green PCR Mastermix (Thermo Fisher Scientific) [10]. No products were detected in any of the viromes after 35 cycles, which does not exclude the presence of contaminating bacterial nucleic acids, but indicates that they were not present at dominant levels. Viral DNA then was amplified using GenomiPhi Hy MDA amplification (GE Healthcare Life Sciences), fragmented to roughly 200 to 400 bp using a Bioruptor (Diagenode), and utilized as input to create libraries using the Ion Plus Fragment Library Kit (Thermo Fisher Scientific) according to manufacturer’s instructions. Libraries then were sequenced using 316 chips on an Ion Torrent Personal Genome Machine (Thermo Fisher Scientific). We trimmed sequence reads according to modified Phred scores of 0.5 using CLC Genomics Workbench 8.5 (Qiagen), removed any low complexity reads with ≥8 consecutive homopolymers, and removed any reads with substantial length variation (<50 nucleotides or >300 nucleotides) or ambiguous characters prior to further analysis. Each virome was screened for contaminating human nucleic acids using BLASTN analysis (E-value <10^−5^) against the human reference database available at ftp://ftp.ncbi.nlm.nih.gov/genomes/H_sapiens/. Any reads with significant sequence similarities to human sequences were removed prior to further analysis using Ion Assist (http://www.thepridelaboratory.org/). All reads were assembled using CLC Genomics Workbench 8.5 (Qiagen) based on 98% identity with a minimum of 50% read overlap, which were more stringent than criteria developed to discriminate between highly related viruses [22]. Because the shortest reads were 50 nucleotides, the minimum tolerable overlap was 25 nucleotides, and the average overlap was no less than 100 nucleotides depending on the characteristics of each virome. The consensus sequence for each contig was constructed according to majority rule, and any contigs <200 nucleotides or with ambiguous characters were removed prior to further analysis. Contigs were annotated using BLASTX against the NCBI NR database with an E-value cutoff value of 10^−5^. Specific viral sequences were identified using Ion Assist (http://www.thepridelaboratory.org/) by parsing BLASTX results for known viral genes including replication, structural, transposition, restriction/modification, hypothetical, and other genes previously found in viruses for which the E-value was at least 10^−5^. Each individual virome contig was annotated using this technique; however, if the best hit for any portion of the contig was to a gene with no known function, lower level hits were used as long as they had known function and still met the E-value cutoff. The sequences of contig71 were generated by PCR amplification of overlapping fragments using Platinum PCR SuperMix (Thermo Fisher Scientific) with specific primers (Additional file 1: Table S3). Each resulting amplicon was in both directions using Sanger sequencing. Contigs were assembled interactively using Sequencher (Gene Codes Corp), ORFs predicted using FGenesV (Softberry Inc, Mount Kisco, NY), and ORF putative functions assigned by BLASTP homology (Escore <10^−5^). If the best hit was to a gene with no known function, lower level hits were used for the annotation as long as they had known putative function and still met the E-score cutoff (10^−5^). Polymorphisms were identified using day 0 as the index sequence.

Analysis of shared sequence similarities present in each virome was performed by creating custom BLAST databases for each virome, comparing each database with all other viromes using BLASTN analysis (E-value <10^−10^), and these compiled data used to calculate Sorensen distances using Ion Assist (http://www.thepridelaboratory.org/). Sorensen distances are measured on a scale of 0 to 1, where 0 represents no sharing and 1 would represent identical viromes. These distances were determined for all pairs of housemates and compared with distances between subjects from different households. Statistical significance was determined by the Mann–Whitney *U* test using MaxStat Pro (http://www.maxstat.de/). Bray Curtis distances (equivalent to 1 minus Sorensen values) also were determined and used as input for principal coordinates analysis using QIIME [23]. We determined persistence of viruses by constructing global assemblies from all contigs within a subject over time, and using the contribution of each time point to the assemblies to decipher the time points that contributed to the construction of each virus, as has previously been described [12]. We utilized this technique to decipher those contigs that were unique to a subject within a household and those shared between housemates. We also created global assemblies from both subjects within a household to determine the presence/absence of viruses at each subject and time point and identify viruses that may have been transmitted between housemates. We defined any virus that was present in one subject, absent in their housemate, and later appeared in at least two time points in the housemate as a putative transmission. Statistical significance was determined by comparisons between groups by the Mann–Whitney *U* test using MaxStat Pro (http://www.maxstat.de/).

To assess whether viromes had significant overlap within or between subjects, we performed a permutation test using Ion Assist (http://www.thepridelaboratory.org/) based on resampling (10,000 iterations) [13]. We simulated the distribution of the fraction of shared virome homologues from two different time points within individual subjects that were randomly chosen across all time points. For each set, we computed the summed fraction of shared homologues using 1000 random contigs between randomly chosen individual time points within different subjects, and from these computed an empirical null distribution of our statistic of interest (the fraction of shared homologues). The simulated statistics within each subject across all time points were referred to the null distribution of inter-subject comparisons, and the *p* value was computed as the fraction of times the simulated statistic for the each exceeded the observed statistic. We utilized a similar technique to determine whether there was significant overlap between the viromes of the different households.

## Additional files

Additional file 1: Table S1. Study Subjects. **Table S2.** Viral homologues within and between households. **Table S3.** Primer sequences used in this study. (PDF 795 kb)
Additional file 2: Figure S1. Principal coordinates analysis of beta diversity present in the saliva of all households. **Figure S2.** Principal coordinates analysis of beta diversity present in the saliva of all subjects. **Figure S3.** Bar graph (± standard error) demonstrating the relative time intervals between the earliest and latest time point that formed each fecal viral contig. **Figure S4.** Bar graph (± standard error) demonstrating the relative time intervals between the earliest and latest time point that formed each salivary viral contig. **Figure S5.** Bar graphs representing the relative proportions of viromes that are unique or shared within each household. **Figure S6.** Bar graphs representing the mean proportions of viromes (± standard error) that are unique or shared within a household. **Figure S7.** Assembly of contig 39 from the feces in all time points from the subjects in household 1. **Figure S8.** Assembly of contig 142 from the feces in all time points from the subjects in household 2. **Figure S9.** Assembly of contig 41 from the feces in all time points from the subjects in household 3. **Figure S10.** Assembly of contig 44 from the feces in all time points from the subjects in household 6. **Figure S11.** Assembly of contig 71 from the feces in all time points from the subjects in household 7. **Figure S12.** Bar graph representing the mean proportions of salivary viromes (± standard error) shared between housemates and putative transmissions.

## Abbreviations

PCOA: Principal coordinates analysis

## References

[1] Hannigan GD, Meisel JS, Tyldsley AS, Zheng Q, Hodkinson BP, SanMiguel AJ, Minot S, Bushman FD, Grice EA. The human skin double-stranded dna virome: topographical and temporal diversity, genetic enrichment, and dynamic associations with the host microbiome. MBio. 2015;6:e01578-15. 10.1128/mBio.01578-15 PMC4620475 26489866

[2] Pride DT, Salzman J, Haynes M, Rohwer F, Davis-Long C, White RA, Loomer P, Armitage GC, Relman DA (2012). Evidence of a robust resident bacteriophage population revealed through analysis of the human salivary virome. ISME J.

[3] Minot S, Sinha R, Chen J, Li H, Keilbaugh SA, Wu GD, Lewis JD, Bushman FD (2011). The human gut virome: inter-individual variation and dynamic response to diet. Genome Res.

[4] Santiago-Rodriguez TM, Ly M, Bonilla N, Pride DT (2015). The human urine virome in association with urinary tract infections. Front Microbiol.

[5] Willner D, Furlan M, Schmieder R, Grasis JA, Pride DT, Relman DA, Angly FE, McDole T, Mariella RP, Rohwer F, Haynes M (2011). Metagenomic detection of phage-encoded platelet-binding factors in the human oral cavity. Proc Natl Acad Sci U S A.

[6] Ly M, Abeles SR, Boehm TK, Robles-Sikisaka R, Naidu M, Santiago-Rodriguez T, Pride DT (2014). Altered oral viral ecology in association with periodontal disease. MBio.

[7] Norman JM, Handley SA, Baldridge MT, Droit L, Liu CY, Keller BC, Kambal A, Monaco CL, Zhao G, Fleshner P (2015). Disease-specific alterations in the enteric virome in inflammatory bowel disease. Cell.

[8] Lewis JD, Chen EZ, Baldassano RN, Otley AR, Griffiths AM, Lee D, Bittinger K, Bailey A, Friedman ES, Hoffmann C (2015). Inflammation, antibiotics, and diet as environmental stressors of the gut microbiome in pediatric Crohn’s disease. Cell Host Microbe.

[9] Modi SR, Lee HH, Spina CS, Collins JJ (2013). Antibiotic treatment expands the resistance reservoir and ecological network of the phage metagenome. Nature.

[10] Abeles SR, Ly M, Santiago-Rodriguez TM, Pride DT (2015). Effects of long term antibiotic therapy on human oral and fecal viromes. PLoS One.

[11] Enault F, Briet A, Bouteille L, Roux S, Sullivan MB, Petit MA. Phages rarely encode antibiotic resistance genes: a cautionary tale for virome analyses. ISME J. 2016;10. doi:10.1038/ismej.2016.90. [Epub ahead of print] 10.1038/ismej.2016.90 PMC5315482 27326545

[12] Abeles SR, Robles-Sikisaka R, Ly M, Lum AG, Salzman J, Boehm TK, Pride DT (2014). Human oral viruses are personal, persistent and gender-consistent. ISME J.

[13] Robles-Sikisaka R, Ly M, Boehm T, Naidu M, Salzman J, Pride DT. Association between living environment and human oral viral ecology. ISME J. 2013;7(9): 1710-24. 10.1038/ismej.2013.63 PMC3749502 23598790

[14] Rothberg JM, Hinz W, Rearick TM, Schultz J, Mileski W, Davey M, Leamon JH, Johnson K, Milgrew MJ, Edwards M (2011). An integrated semiconductor device enabling non-optical genome sequencing. Nature.

[15] Dutilh BE, Cassman N, McNair K, Sanchez SE, Silva GG, Boling L, Barr JJ, Speth DR, Seguritan V, Aziz RK (2014). A highly abundant bacteriophage discovered in the unknown sequences of human faecal metagenomes. Nat Commun.

[16] Song SJ, Lauber C, Costello EK, Lozupone CA, Humphrey G, Berg-Lyons D, Caporaso JG, Knights D, Clemente JC, Nakielny S (2013). Cohabiting family members share microbiota with one another and with their dogs. Elife.

[17] Lax S, Smith DP, Hampton-Marcell J, Owens SM, Handley KM, Scott NM, Gibbons SM, Larsen P, Shogan BD, Weiss S (2014). Longitudinal analysis of microbial interaction between humans and the indoor environment. Science.

[18] Kleiner M, Hooper LV, Duerkop BA (2015). Evaluation of methods to purify virus-like particles for metagenomic sequencing of intestinal viromes. BMC Genomics.

[19] Abeles SR, Jones MB, Santiago-Rodriguez TM, Ly M, Klitgord N, Yooseph S, Nelson KE, Pride DT (2016). Microbial diversity in individuals and their household contacts following typical antibiotic courses. Microbiome.

[20] Willner D, Furlan M, Haynes M, Schmieder R, Angly FE, Silva J, Tammadoni S, Nosrat B, Conrad D, Rohwer F (2009). Metagenomic analysis of respiratory tract DNA viral communities in cystic fibrosis and non-cystic fibrosis individuals. PLoS One.

[21] Murphy FA, Fauquet CM, Bishop DHL, Ghabrial SA, Jarvis AW, Martelli GP, Mayo MA, Summers MD (1995). Virus taxonomy: sixth report of the International Committee on Taxonomy of Viruses.

[22] Breitbart M, Salamon P, Andresen B, Mahaffy JM, Segall AM, Mead D, Azam F, Rohwer F (2002). Genomic analysis of uncultured marine viral communities. Proc Natl Acad Sci U S A.

[23] Caporaso JG, Kuczynski J, Stombaugh J, Bittinger K, Bushman FD, Costello EK, Fierer N, Pena AG, Goodrich JK, Gordon JI (2010). QIIME allows analysis of high-throughput community sequencing data. Nat Meth.

