# Supplementary material for: Transmission of viruses via our microbiomes
Source: Microbiome. 2016 Dec 2;4:64. doi: 10.1186/s40168-016-0212-z (PMC5134127; doi:10.1186/s40168-016-0212-z)
Supplement: Additional file 1: Table S1. — Study Subjects. Table S2. Viral homologues within and between households. Table S3. Primer sequences used in this study. (PDF 795 kb) [file 40168_2016_212_MOESM1_ESM.pdf]

Table S1. Study Subjects

	Subject	Age	Sex	Ethnicity	Antibiotic	Vegetarian	Couple or Roommate
House 1							
	CA05	23	F	Caucasian	Amoxicillin	No	Couple
	CA06	24	M	Caucasian	None	No	Couple
House 2							
	CA13	31	F	Caucasian	Amoxicillin	No	Couple
	CA14	27	F	Caucasian	None	No	Couple
House 3							
	CA23	34	M	Caucasian	Amoxicillin	No	Couple
	CA24	21	M	Caucasian	None	No	Couple
House 4							
	CA27	24	M	Caucasian	Amoxicillin	No	Couple
	CA28	24	F	Caucasian	None	No	Couple
House 5							
	CA11	19	F	Asian	Azithromycin	No	Couple
	CA12	20	M	Latino	None	No	Couple
House 6							
	CA15	24	M	Asian	Azithromycin	No	Roommate
	CA16	21	M	Asian	None	No	Roommate
House 7							
	CA39	63	M	Asian	Azithromycin	No	Roommate
	CA40	60	M	Latino	None	No	Roommate
House 8							
	CA47	24	F	Caucasian	Azithromycin	No	Couple
	CA48	25	M	Caucasian	None	No	Couple
Controls							
	CA81	19	F	Caucasian	None	No	Neither
	CA85	21	M	Asian	None	No	Neither
	CA87	28	F	Latino	None	No	Neither
	CA90	19	F	Asian	None	No	Neither

Table S2. Viral homologues within and between households

By Subject	Percent Homologous within Household[a]	Percent Homologous between Households[a]	P value[b]
**Feces**			
House 1	32.77 ± 7.80	5.25 ± 5.60	**0.0039**
House 2	18.16 ± 5.27	3.90 ± 3.78	**0.0163**
House 3	34.43 ± 5.43	1.50 ± 1.84	**<0.0001**
House 4	6.37 ± 5.80	2.04 ± 2.92	0.2219
House 5	17.63 ± 5.12	4.58 ± 4.43	**0.0454**
House 6	15.41 ± 3.94	5.68 ± 7.43	0.0600
House 7	27.12 ± 22.45	4.68 ± 7.49	0.0808
House 8	38.86 ± 10.27	5.50 ± 4.94	**0.0030**
**Saliva**			
House 1	17.30 ± 7.19	2.46 ± 4.27	**0.0298**
House 2	14.21 ± 9.55	6.77 ± 8.36	0.2336
House 3	12.60 ± 12.95	4.90 ± 6.87	0.2733
House 4	31.91 ± 18.39	4.47 ± 6.28	**0.0501**
House 5	26.64 ± 5.38	7.28 ± 7.61	**0.0192**
House 6	21.79 ± 17.21	7.04 ± 9.42	0.1409
House 7	7.33 ± 3.70	4.04 ± 6.28	0.2126
House 8	26.12 ± 15.57	9.08 ± 9.98	0.1512

[a]Based on the mean of 10,000 iterations. 10,000 random contigs were sampled per iteration

[b]Empirical p-value based on the fraction of times the estimated percent homologous reads within each household exceeds that for different households.

Table S3: Primer sequences used in this study

Primer Name	Primer Sequence (5'-3')
Contig71-13F	GTACTTATTGCCAACCATTG
Contig71-392R	CATTAGAGTACAAATCCC
Contig71-370F	TCTCAGGGATTTGTACTC
Contig71-754R	CTGCTTCCTTAGCTTCATT
Contig71-739F	GAAGCTAAGGAAGCAGTAA
Contig71-1188R	TGCTTTGGTTAACTGGTC
Contig71-1173F	CCAGTTAACCAAAGCAACA
Contig71-1732R	CCACCTTTAAATTCTCCC
Contig71-1711F	GCATGGGAGAATTTAAAGG
Contig71-2110R	CATATACTACACCACCAAC
Contig71-2091F	AGTTGGTGGTGTAGTATATG
Contig71-2480R	GCTTTTTTCCTTTCTTCCTC
Contig71-2461F	GAGGAAGAAAGGAAAAAAGC
Contig71-2876R	ACCCCACCAAATTCATATTC
Contig71-2867F	TTGGTGGGGTAATAAGGG
Contig71-3394R	TCTAAAGAACAGGTGGGG
Contig71-3376F	GCCCCACCTGTTCTTTAG
Contig71-3911R	ACCCAATATCCAGAATCACC
Contig71-3893F	GTGATTCTGGATATTGGGTT
Contig71-4461R	GGCCAGTTGGATTATTGT
Contig71-4476F	TACCGTGAAATACCTATCC
Contig71-4918R	GTTAACTTACCATCTCTACC
Contig71-4894F	TGGATGGTAGAGATGGTAAG
Contig71-5654R	TGGGTTTGGATTACGTGG
Contig71-5639F	ACGTAATCCAAACCCACAA
Contig71-6172R	CCAGCTTTTTCTGCTTCTC
Contig71-6183F	GGCACTGCTAGGTCAAAA
Contig71-6588R	CCATGAAAACAACCTCCAA
Contig71-6631F	AGAATTTGAGGGTGGTGA
Contig71-7201R	GACCGGTAGTATATGTAATG
Contig71-7195F	ACCGGTCTAGTAACTTCA
Contig71-7605R	GGCTGTTATCCTTGGTTC
Contig71-7587F	GGAACCAAGGATAACAGC
Contig71-8269R	TTGGTCTGAGGGTATATCG
Contig71-8251F	CGATATACCCTCAGACCA
Contig71-8595R	TCATAGTCAGAACCACCA
Contig71-8578F	TGGTGGTTCTGACTATGA
Contig71-9236R	GACCTTCCCCATAGAATTT
Contig71-9219F	AATTCTATGGGGAAGGTC
Contig71-9673R	ATATCAGGGGTAACATCCA

Contig71-9723F	GGGGTATTAGTAAAAGGTG
Contig71-10314R	AGTTTGGAGTAGGATGGA
Contig71-10299F	CATCCTACTCCAAACTGAA
Contig71-10871R	GAGGTTGATTGGTCCATA
Contig71-10855F	ATGGACCAATCAACCTCT
Contig71-11323R	GCTCTTGCATTTATACCG
Contig71-11337F	GGTGACCCAGATAAGGAAA
Contig71-11678R	CCAAAAACACAAAGAGCAG
Contig71-11535F	TAGTTGAGTTGATGGTGAG
Contig71-11971R	GGAATGCTTATACTAGAC
Contig71-11954F	GTCTAGTATAAGCATTCC
Contig71-12570R	TAGAGTAAAGTAACCTGG
Contig71-12553F	CCAGGTTACTTTACTCTA
Contig71-13088R	GTTTTCTTGGCTTTTGGT
Contig71-12985F	ATAATCCCCACTCCATAC
Contig71-13551R	TCTTTTTGATGCTTCCCT
Contig71-13535F	GGGAAGCATCAAAAAGAA
Contig71-14028R	ATTTACCATCTCTACCAC
Contig71-14007F	TATTGTGGTAGAGATGGT
Contig71-14613R	GGTATCTGGATATTGCTT
Contig71-14566F	TGGGAGAAAAAGGAATTG
Contig71-15151R	GCCCATTGATTATCTGTT
Contig71-15145F	AATGGGCTCAAATAAGGG
Contig71-15606R	GTTGGGCTTTTTCATTTC
Contig71-15577F	TGGAGGATTTGGGAAATG
Contig71-16001R	TTGAGTTGGGATTATTGG
Contig71-15982F	AACCAATAATCCCAACTC
Contig71-16637R	TACCTTTTCCCCTATACC
Contig71-16529F	ATTTATTGGGGATGTGGTAG
Contig71-17112R	TTCTATGATTGCCATGCC
