# Supplementary material for: Transmission of viruses via our microbiomes
Source: Microbiome. 2016 Dec 2;4:64. doi: 10.1186/s40168-016-0212-z (PMC5134127; doi:10.1186/s40168-016-0212-z)
Supplement: Additional file 2: Figure S1. — Principal coordinates analysis of beta diversity present in the saliva of all households. Figure S2. Principal coordinates analysis of beta diversity present in the saliva of all subjects. Figure S3. Bar graph (± standard error) demonstrating the relative time intervals between the earliest and latest time point that formed each fecal viral contig. Figure S4. Bar graph (± standard error) demonstrating the relative time intervals between the earliest and latest time point that formed each salivary viral contig. Figure S5. Bar graphs representing the relative proportions of viromes that are unique or shared within each household. Figure S6. Bar graphs representing the mean proportions of viromes (± standard error) that are unique or shared within a household. Figure S7. Assembly of contig 39 from the feces in all time points from the subjects in household 1. Figure S8. Assembly of contig 142 from the feces in all time points from the subjects in household 2. Figure S9. Assembly of contig 41 from the feces in all time points from the subjects in household 3. Figure S10. Assembly of contig 44 from the feces in all time points from the subjects in household 6. Figure S11. Assembly of contig 71 from the feces in all time points from the subjects in household 7. Figure S12. Bar graph representing the mean proportions of salivary viromes (± standard error) shared between housemates and putative transmissions. [file 40168_2016_212_MOESM2_ESM.pdf]

Figure S1

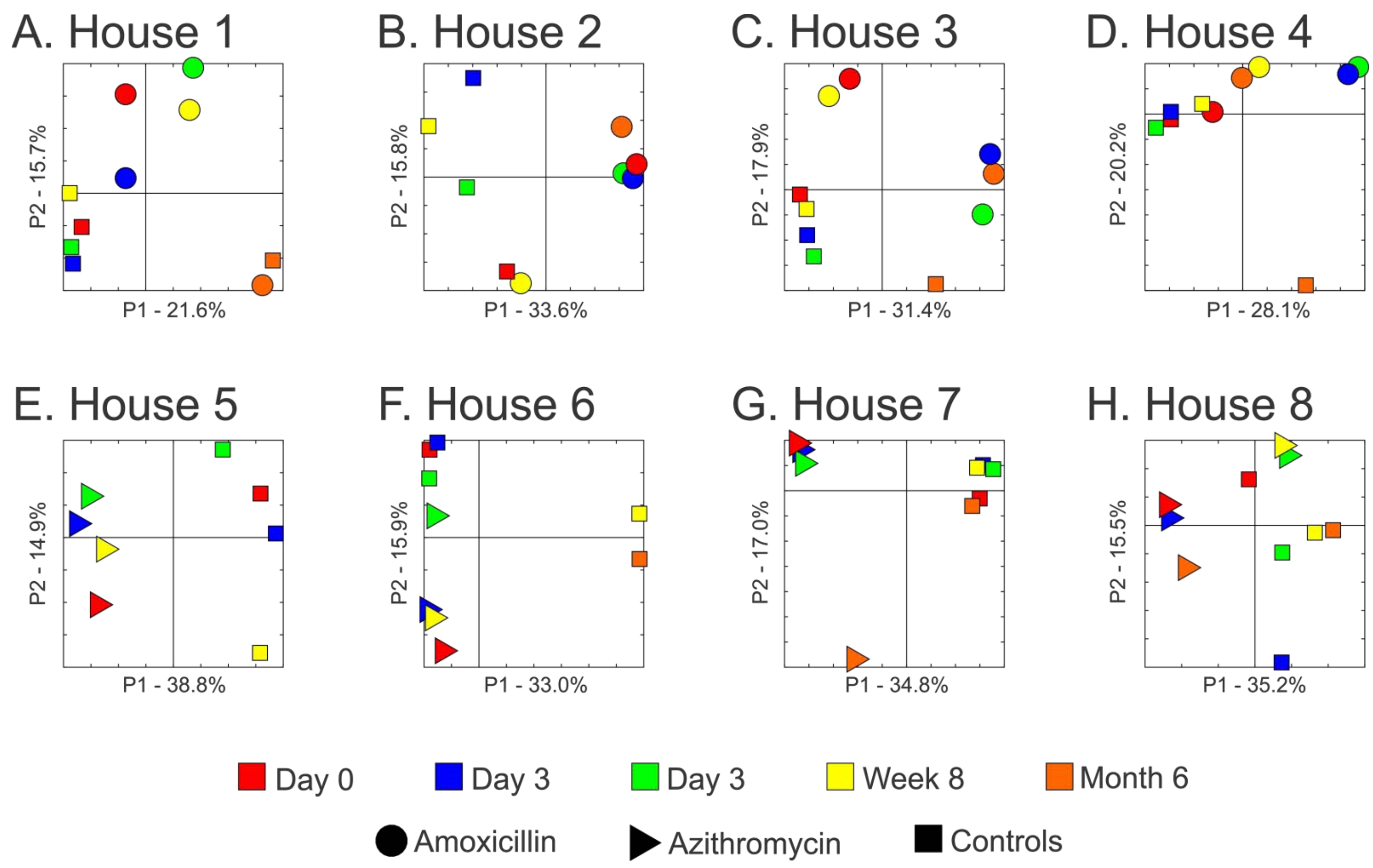

Figure S1: Principal coordinates analysis of beta diversity present in the saliva of all households.
Panels A-H represent households 1-8 respectively. Subjects who received placebo are represented by squares, amoxicillin are represented by circles, and azithromycin are represented by triangles. Specimens collected from Day 0 are represented in red, Day 3 in blue, Day 7 in green, Week 8 in yellow, and Month 6 in orange.

Figure S2

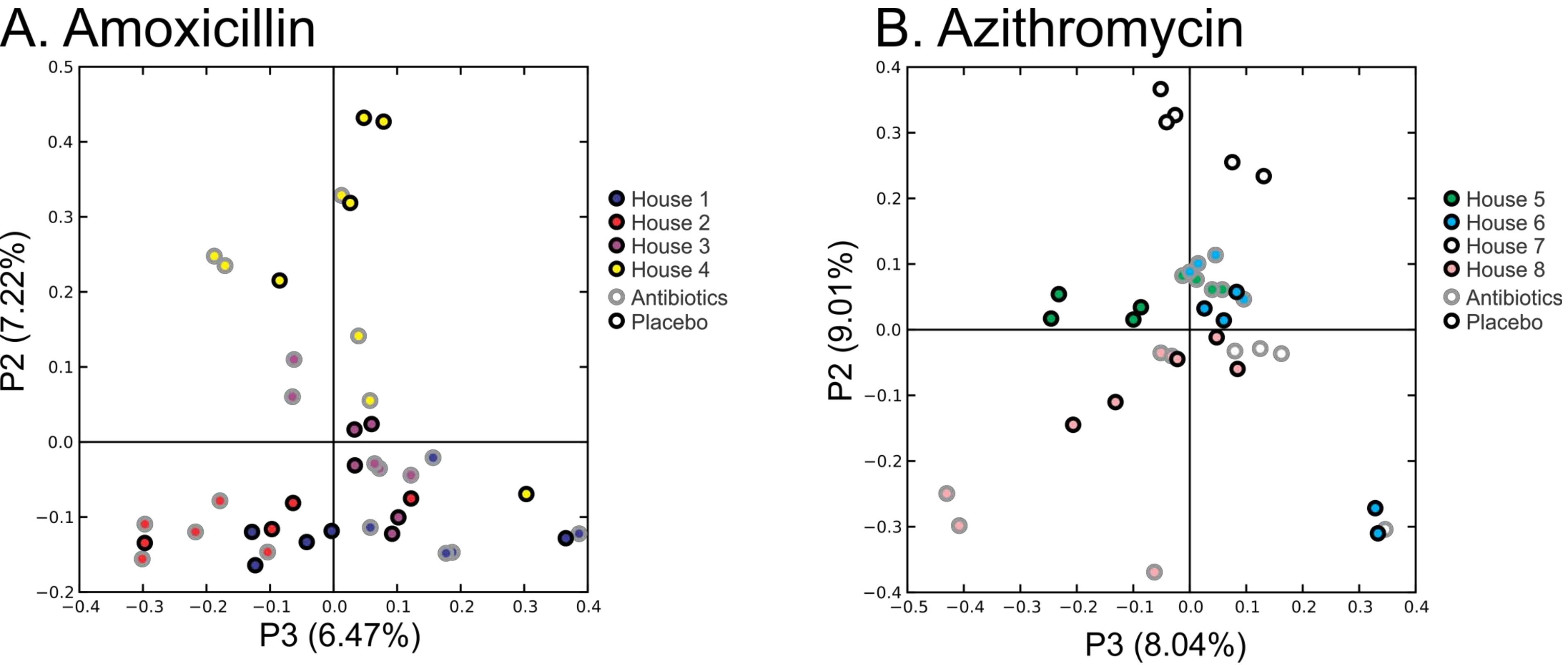

Figure S2: Principal coordinates analysis of beta diversity present in the saliva of all subjects. Panel A represents subjects who received amoxicillin, their housemates who received a placebo. Panel B represents subjects who received amoxicillin and their housemates who received a placebo. Controls who received placebo are demonstrated by black outer circles and subjects who received antibiotics are represented by gray outer circles.

Figure S3

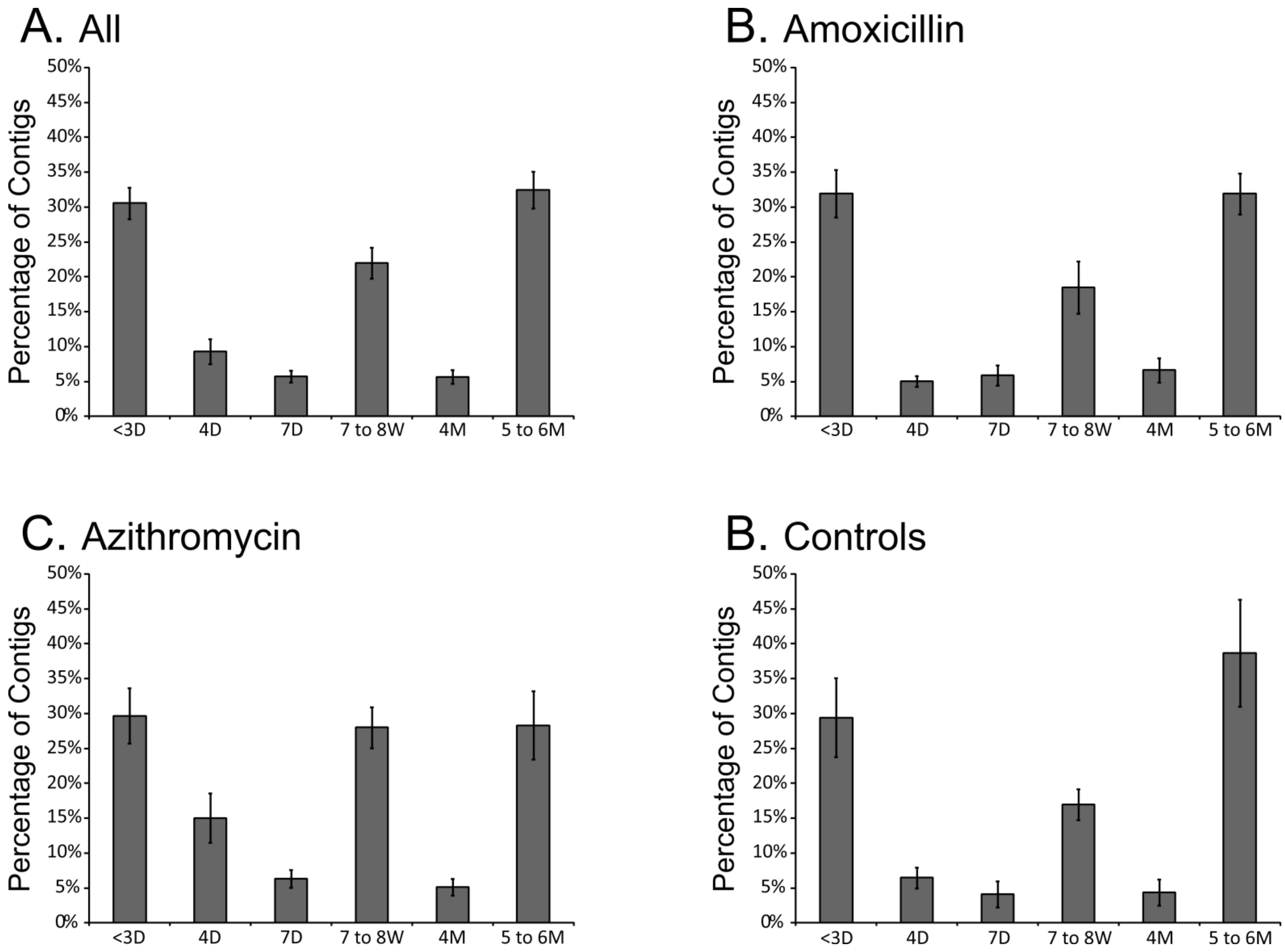

Figure S3: Bar graph (± standard error) demonstrating the relative time intervals between the earliest and latest time point that formed each fecal viral contig. Panel A represents all subjects, Panel B represents households that took amoxicillin (including subjects in those households that took placebo), Panel C represents households that took azithromycin (including subjects in those households that took placebo), and Panel D represents control subjects who took no therapy.

Figure S4

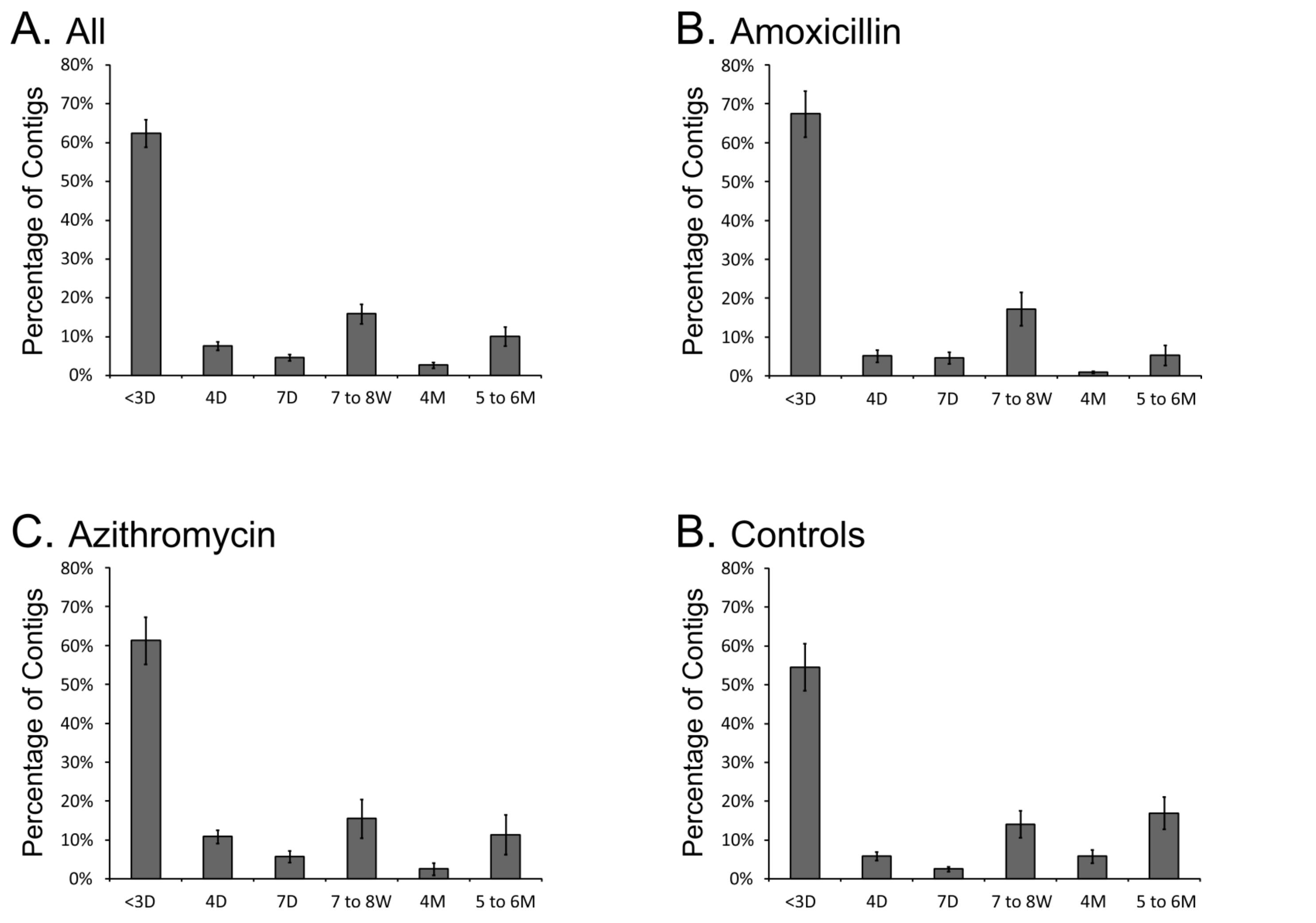

Figure S4: Bar graph (± standard error) demonstrating the relative time intervals between the earliest and latest time point that formed each salivary viral contig. Panel A represents all subjects, Panel B represents households that took amoxicillin (including subjects in those households that took placebo), Panel C represents households that took azithromycin (including subjects in those households that took placebo), and Panel D represents control subjects who took no therapy.

Figure S5

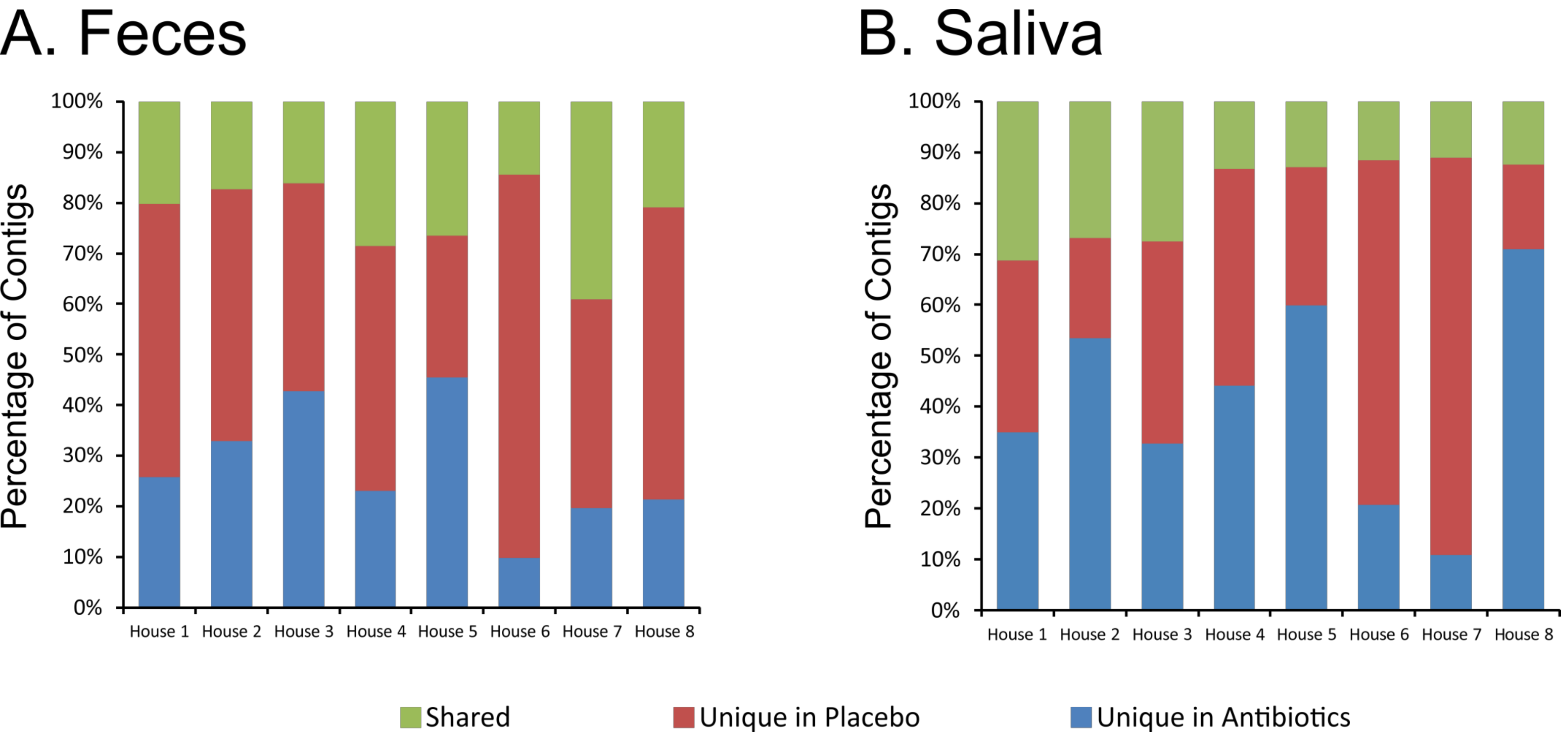

Figure S5: Bar graphs representing the relative proportions of viromes (± standard error) that are unique to subjects taking antibiotics in each household, unique to subjects taking placebo in each household, and shared between the subjects in each household. Panel A represents viruses in feces, and Panel B represents viruses in saliva.

Figure S6

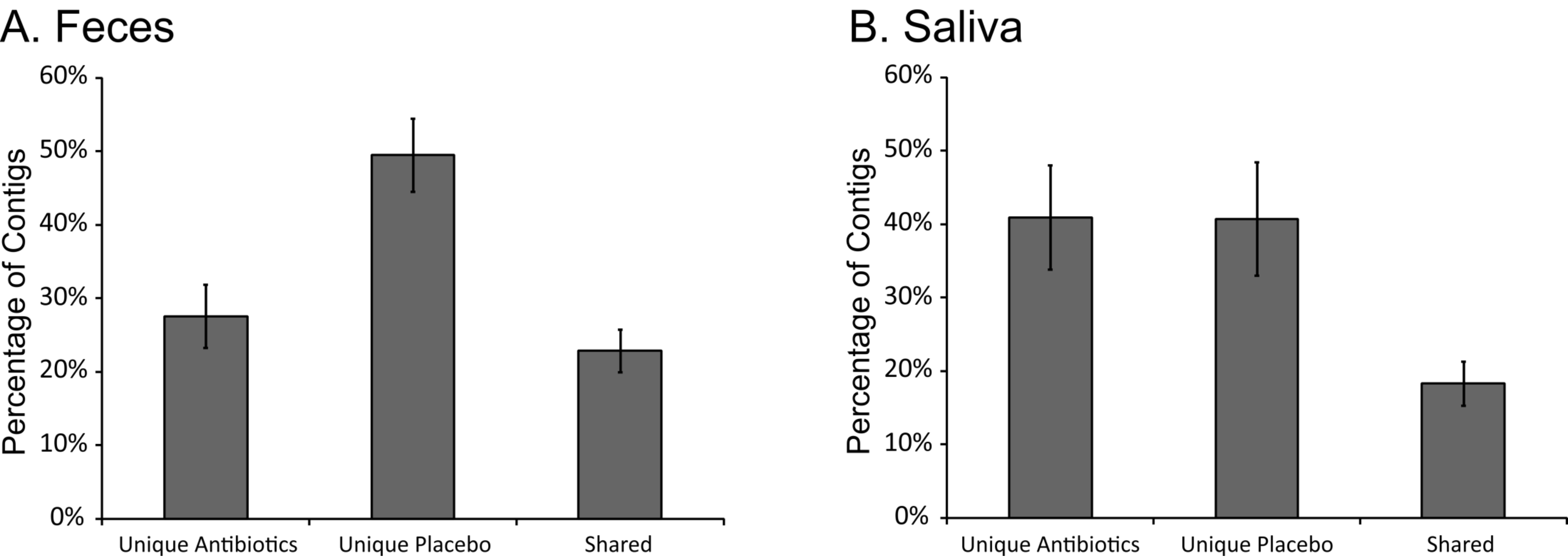

Figure S6: Bar graphs representing the relative proportions of viromes (± standard error) that are unique to subjects taking antibiotics in each household, unique to subjects taking placebo in each household, and shared between the subjects in each household. Panel A represents viruses in feces, and Panel B represents viruses in saliva.

Figure S7

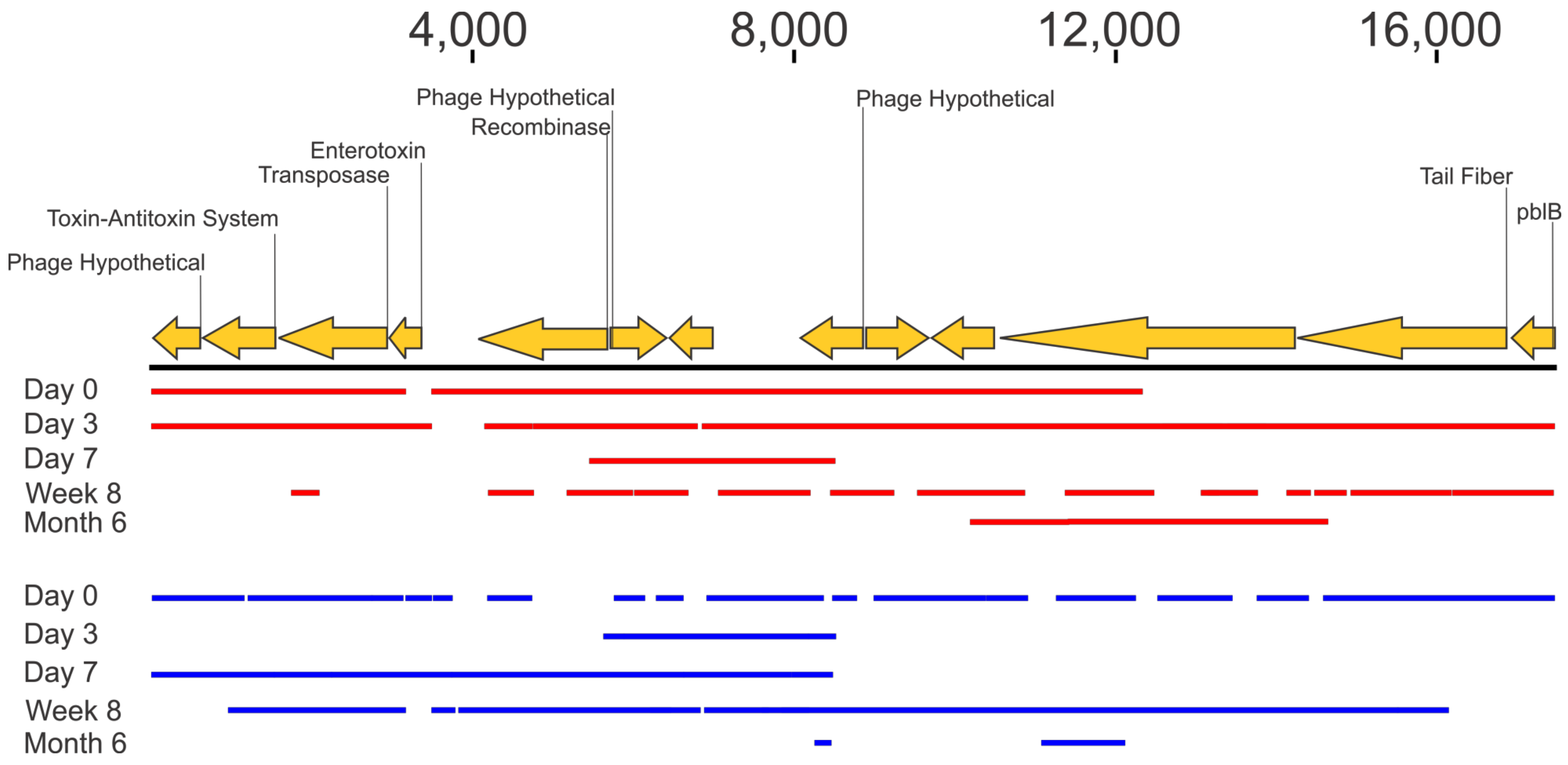

Figure S7: Assembly of contig 39 from the feces in all time points from the subjects in household 1. The portions of the contig identified in each time point are represented by the colored boxes. Putative ORFs and their directions are represented by the yellow arrows and their annotations are represented above. The length of the contig is denoted at the top.

Figure S8

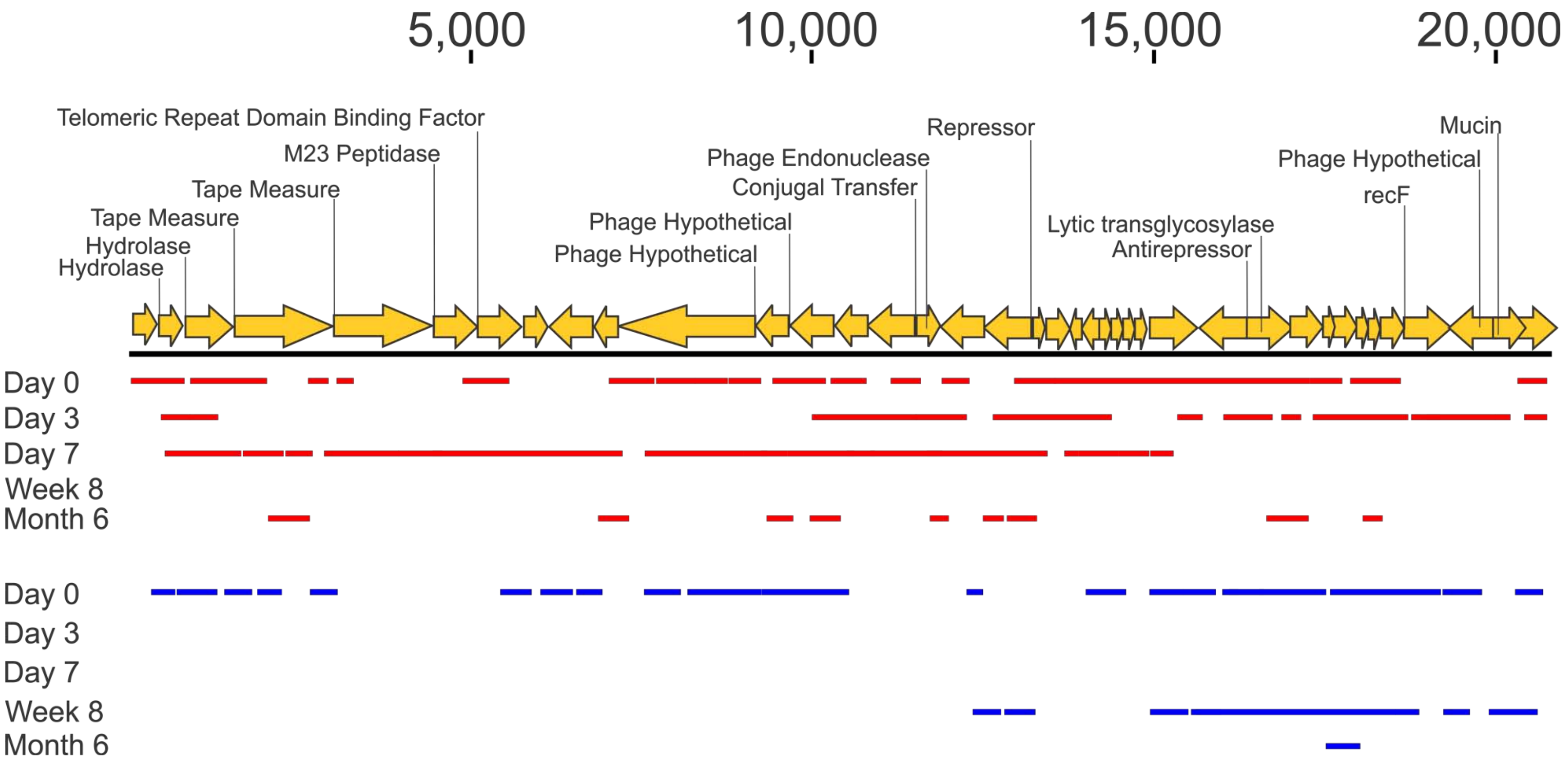

Figure S8: Assembly of contig 142 from the feces in all time points from the subjects in household 2. The portions of the contig identified in each time point are represented by the colored boxes. Putative ORFs and their directions are represented by the yellow arrows and their annotations are represented above. The length of the contig is denoted at the top.

Figure S9

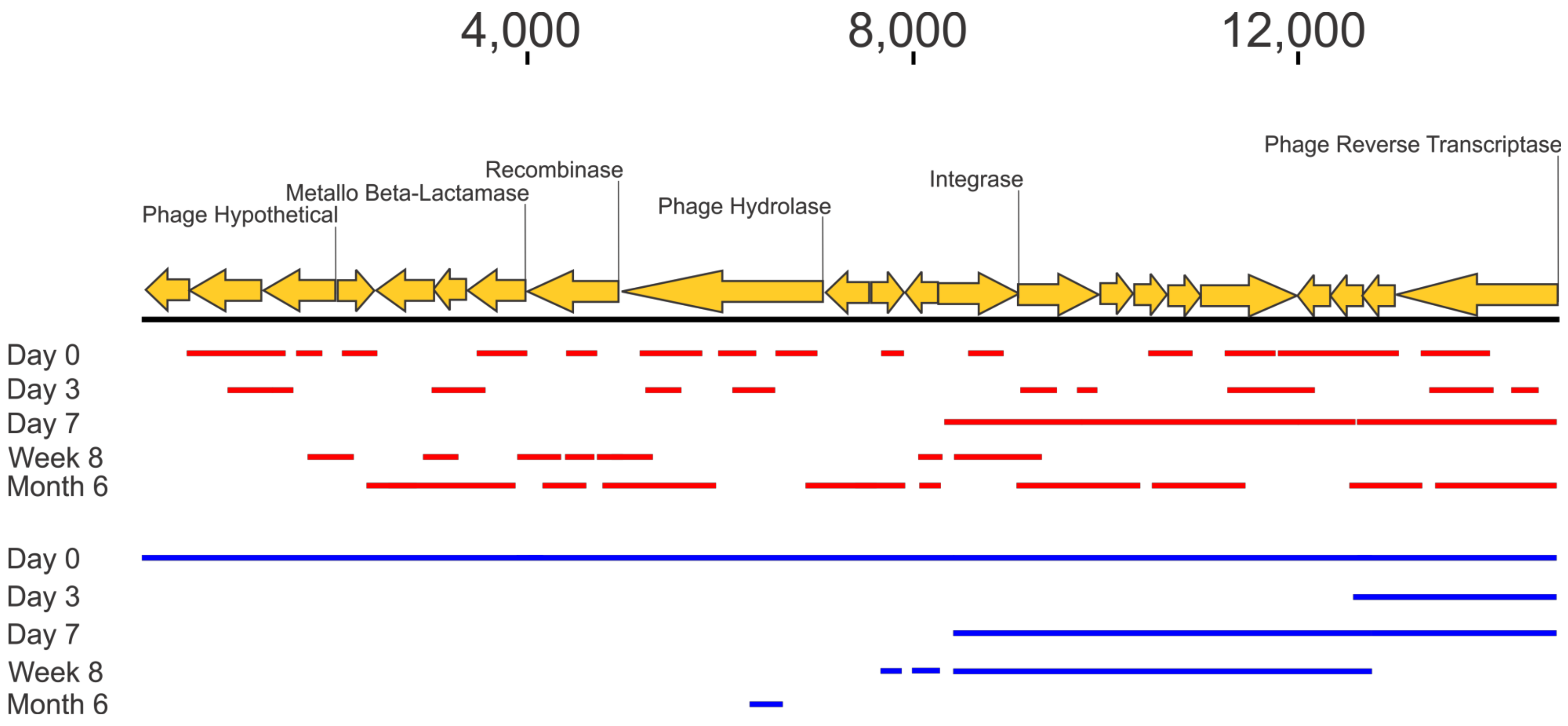

Figure S9: Assembly of contig 41 from the feces in all time points from the subjects in household 3. The portions of the contig identified in each time point are represented by the colored boxes. Putative ORFs and their directions are represented by the yellow arrows and their annotations are represented above. The length of the contig is denoted at the top.

Figure S10

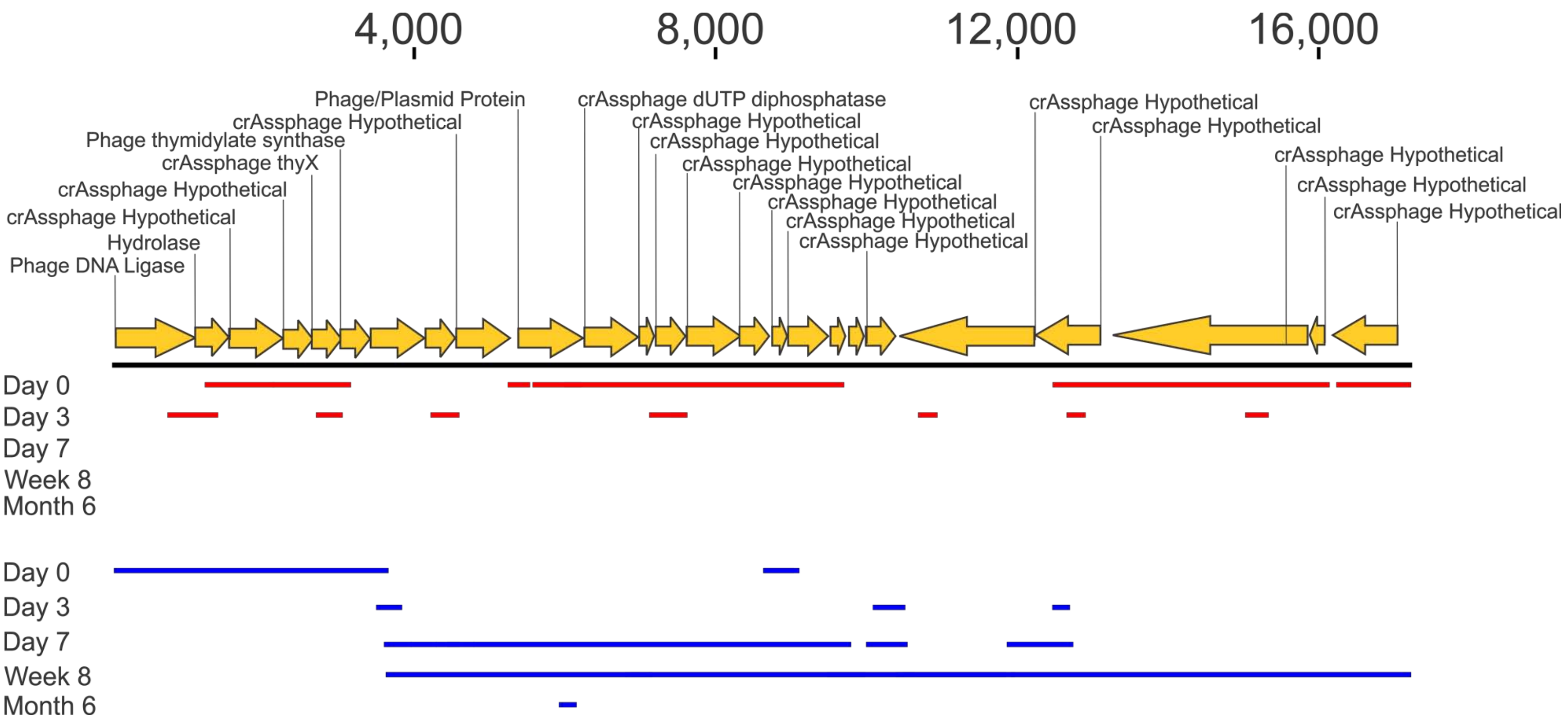

Figure S10: Assembly of contig 44 from the feces in all time points from the subjects in household 6. The portions of the contig identified in each time point are represented by the colored boxes. Putative ORFs and their directions are represented by the yellow arrows and their annotations are represented above. The length of the contig is denoted at the top.

Figure S11

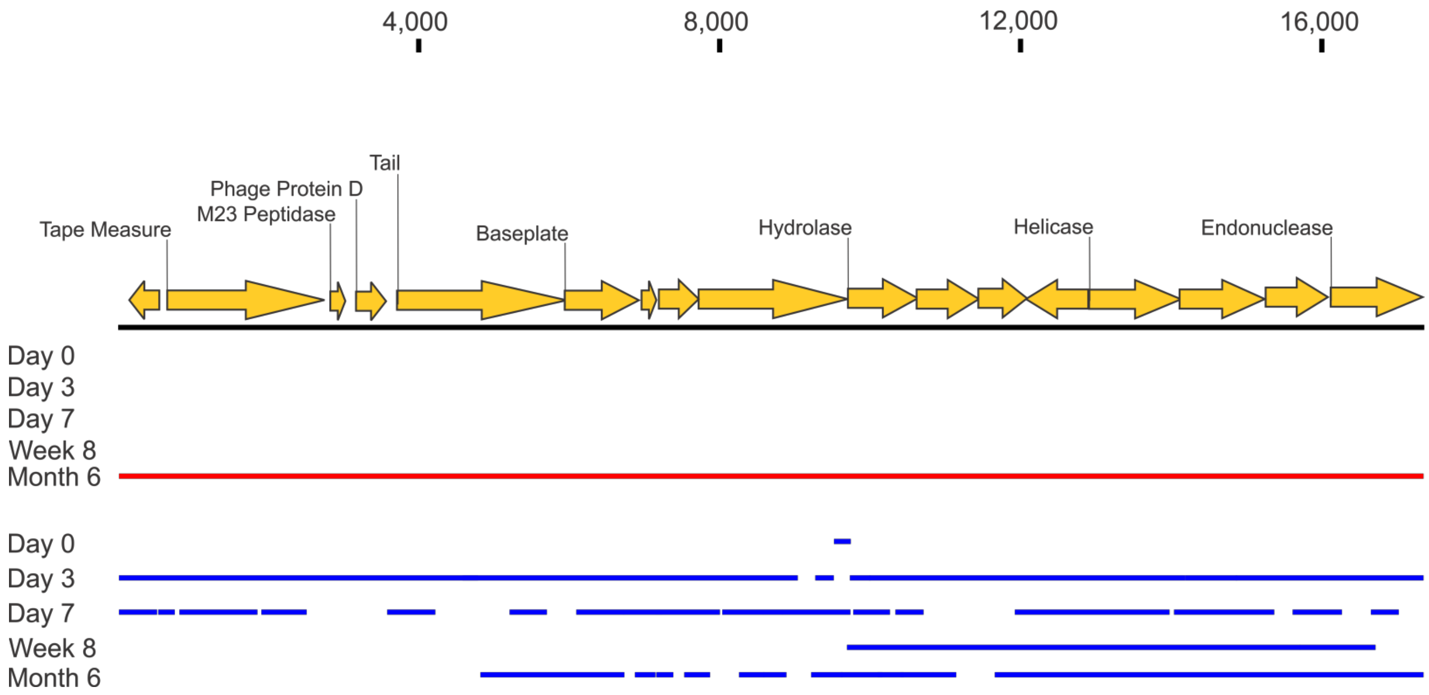

Figure S11: Diagram of contig71 assembled from Sanger sequences from all time points in subject CA40 and from the Month 6 time point in CA39. The contig was not identified on days 0, 3, 7, or week 8 in CA39. Putative ORFs and their direction are indicated by the arrows at the top of the diagram. ORFs that had significant homologues (BLASTP E-score $<10^{-5}$) are indicated by the text above each arrow. The location of polymorphisms (when compared to the day 0 time point in CA40) are indicated by orange vertical lines.

Figure S12

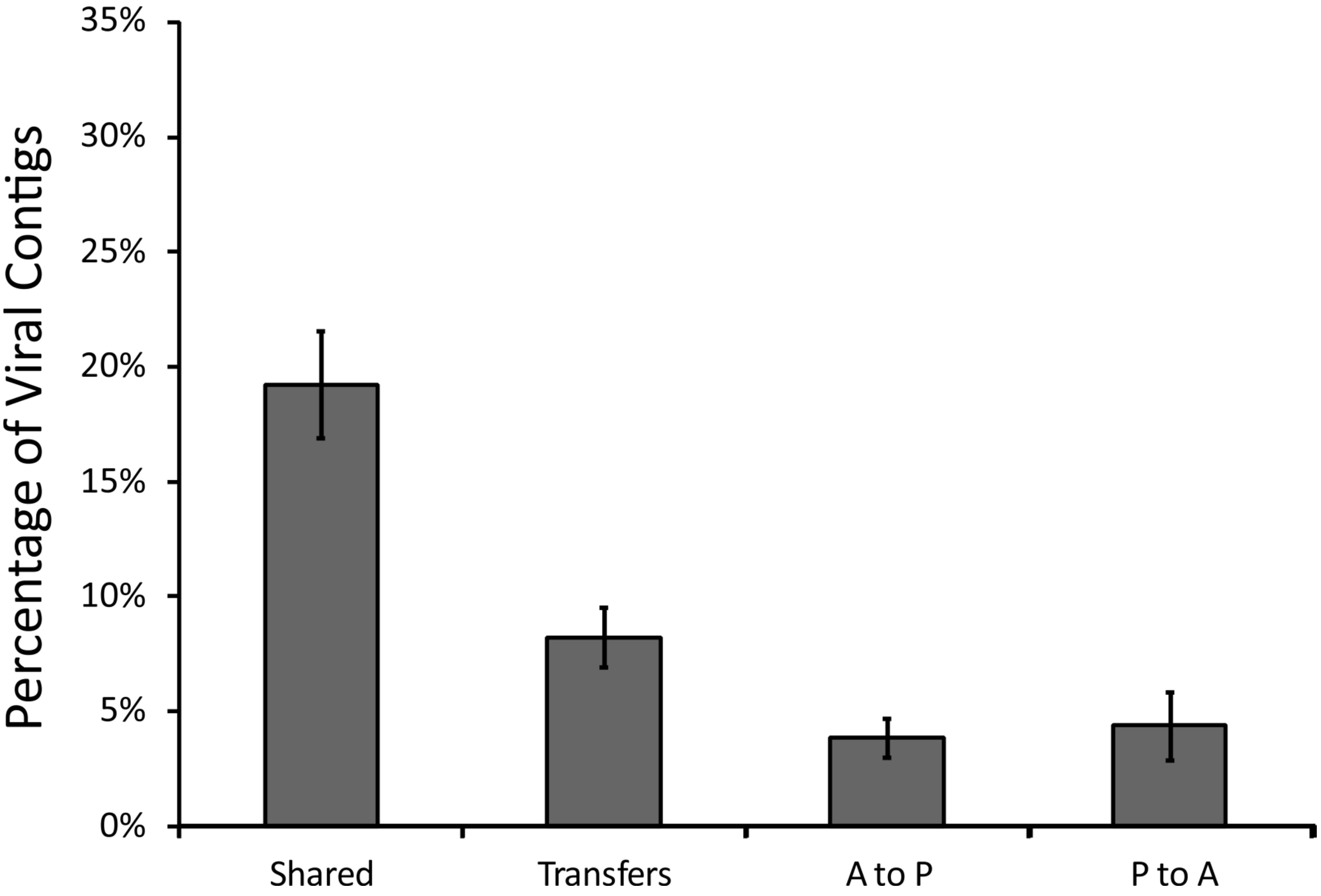

Figure S12: Bar graph representing the mean proportions of salivary viromes (± standard error) shared between housemates, putative transmissions between housemates, putative transmissions from subjects taking antibiotics to subjects taking placebo, and putative transmissions from subjects taking placebo to subjects taking antibiotics.
